# Contemporary Grading of Prostate Cancer: The Impact of Grading Criteria and the Significance of the Amount of Intraductal Carcinoma

**DOI:** 10.3390/cancers13215454

**Published:** 2021-10-29

**Authors:** Vasiliki Tzelepi, Ioanna Maria Grypari, Souzana Logotheti, Stavros Kontogiannis, Panagiotis Kallidonis, Maria Melachrinou, Vasiliki Zolota

**Affiliations:** 1 Department of Pathology, School of Medicine, University of Patras, 26504 Patras, Greece; med5812@upnet.gr (I.M.G.); slogotheti@upnet.gr (S.L.); melachrinou@upatras.gr (M.M.); vickyzolota@upatras.gr (V.Z.); 2 Department of Pathology, University Hospital of Patras, 26504 Patras, Greece; 3 Department of Urology, School of Medicine, University of Patras, 26504 Patras, Greece; kontogstas@upatras.gr (S.K.); pkallidonis@upatras.gr (P.K.)

**Keywords:** prognostic grade group, Gleason Score, intraductal carcinoma, cribriform pattern

## Abstract

**Simple Summary:**

Prognostic grade group is an important prognostic parameter in prostate cancer, guiding therapeutic decisions. The cribriform pattern and intraductal carcinoma are histologic patterns with additional prognostic significance and their presence should be commented upon in pathology reports. The cribriform pattern is included in grade grouping. Controversies exist regarding the grading of intraductal carcinoma. The grading of tertiary patterns is another point of disagreement in the recently published guidelines. In this study, we sought to address the impact of the different guidelines in prostate cancer grading of prostatectomy specimens. The association of the amount of intraductal carcinoma to pathologic parameters was also analyzed. Our study highlights the potential of confusion among pathologists and clinicians in regard to prostate cancer grading and underscores the need for a consensus grading system.

**Abstract:**

(1) Background: Prognostic grade group (PGG) is an important prognostic parameter in prostate cancer that guides therapeutic decisions. The cribriform pattern and intraductal carcinoma (IDC) are two histological patterns, that have additional prognostic significance. However, discrepancies exist regarding the handling of IDC according to the guidelines published by two international genitourinary pathology societies. Furthermore, whether, in addition to its presence, the amount of IDC is also of importance has not been studied before. Lastly, the handling of tertiary patterns has also been a matter of debate in the literature. (2) Methods: A total of 129 prostatectomy cases were retrieved and a detailed histopathologic analysis was performed. (3) Results: Two cases (1.6%) upgraded their PGG, when IDC was incorporated in the grading system. The presence and the amount of IDC, as well as the presence of cribriform carcinoma were associated with adverse pathologic characteristics. Interestingly, in six cases (4.7%) there was a difference in PGG when using the different guidelines regarding the handling of tertiary patterns. In total, 6.2% of the cases would be assigned a different grade depending on the guidelines followed. (4) Conclusions: These findings highlight a potential area of confusion among pathologists and clinicians and underscore the need for a consensus grading system.

## 1. Introduction

Gleason grade was proposed in 1974 by Donald Gleason [1] and, after several fundamental modifications [2,3], remains one of the most powerful prognostic factors in prostate cancer (PCa). In 2013, a new grading system named (prognostic) grade group (PGG) was proposed by Epstein et al. [4]. Subsequently, PGG was adopted by the International Society of Pathology (ISUP) and included in the 2016 WHO Classification of Prostate tumors publication [5]. In the new system, tumors with a GS of 6 or less are designated as PGG1, those with GS 7 (3 + 4) as PGG2, those with GS 7 (4 + 3) as PGG3, those with GS 8 (with any combination) as PGG4 and those with GS 9 (4 + 5, 5 + 4) and 10 as PGG5. Several studies have proven the value of this system [6,7] in stratifying prostate cancer patients and current guidelines suggest that both the Gleason Score and PGG are reported by Pathologists.

Two patterns of PCa seem to have additional prognostic significance; that is the cribriform pattern and intraductal carcinoma. The cribriform pattern is defined as a continuous proliferation of cells with intermingled lumina and is assigned a Gleason Grade (GG) 4 (or 5 if associated with comedo necrosis) [3,8,9]. The presence of a cribriform pattern in a tumor has been associated with adverse pathologic parameters [10,11] and earlier PSA recurrence [12,13]. Additionally, it has been shown that PCa assigned a GS 7 without a cribriform pattern on biopsy has a similar prognosis with a tumor assigned a GS 6, giving the opportunity for active surveillance to these patients [11]. According to the most recent guidelines, the presence and significance of a cribriform pattern, although already considered in grading, should be commented upon in biopsies and prostatectomy specimens [14,15]. 

Intraductal carcinoma is defined as ‘an intra-acinar and/or intraductal neoplastic proliferation that has some features of high-grade prostatic intraepithelial neoplasia but exhibits much greater architectural and/or cytological atypia’ [5]. Histologically, IDC is characterized by a solid/dense cribriform pattern or a loose cribriform/micropapillary pattern with either marked nuclear atypia (nuclear size ≥6 times the size of the normal cells) or comedo necrosis [16]; however, the criterion of nuclear size has been questioned recently [17,18] and has been replaced by marked pleomorphism/nucleomegaly [14]. IDC is usually accompanied by an invasive high-grade adenocarcinoma [19], even though cases with low grade carcinoma [20,21], or even, absence of invasive carcinoma [22] have been described in both prostate biopsies and prostatectomies. The presence of IDC is associated with adverse pathologic parameters [11,23], and represents an independent predictor of reduced progression-free survival in hormone naïve [24,25], treated [26] and CRPC patients [27]. However, whether in addition to the presence, the amount of IDC present is also of importance has not been studied before. 

Similar to the cribriform pattern, presence of IDC should be commented upon in both biopsies and prostatectomy specimens with a note regarding its aggressive behavior [14,15]. However, controversy exists regarding the grading of IDC in the guidelines published by two international genitourinary pathology societies (ISUP and Genitourinary Pathology Society (GUPS)) [14,15]. Both societies agree that isolated IDC carcinoma (i.e., not accompanied by an invasive neoplasm) should not be graded. However, a different approach is followed when invasive cancer is present. ISUP suggests that IDC of the prostate should be incorporated into PGG and graded as GG4 (or 5 in presence of comedo necrosis) [15], whereas GUPS suggests that IDC should not be included in grading, but be separately reported instead [14]. A separate comment on the clinical significance of the presence of IDC is endorsed by both societies [14,15].

In addition to IDC grading, reporting of tertiary patterns in prostatectomies is another controversy in the literature [28]. A tertiary pattern was originally defined as the third most common pattern seen in a prostate carcinoma. However, in the last years it is being considered synonymous with a minor high-grade pattern, with minor usually being <5%, and its presence has been associated with worse prognosis [29]. ISUP defines a tertiary pattern as a pattern of 4 or 5 that is <5% of the tumor volume and does not incorporate it in PGG [15], whereas GUPS accepts as tertiary only a <5% pattern 5 in tumors with grade group 2 or 3, calling it “minor tertiary 5” [14]. 

These discrepancies in the guidelines of PCa grading can potentially create confusion, not only among pathologists, but also urologists and oncologists as they may not know which system is followed by their pathologist. In fact, in the College of American Pathologists protocols on PCa reporting, a specific note has been included as to whether IDC is incorporated into a grade or not [30,31]. In this study, we sought to examine the impact of the discrepancies in PCa grading according to the different guidelines in prostatectomy specimens. In addition, we analyzed whether not only the presence, but also the amount of IDC would have any significance, as this would support its inclusion in grading.

## 2. Materials and Methods

### 2.1. Patients

The electronic files (that go back up to 2000) of the Department of Pathology of the University Hospital of Patras were searched and all prostatectomies with lymph node metastasis were retrieved from the years 2000–2017. In addition, radical prostatectomy specimens with lymph node dissection and absence of lymph node metastasis (N0) were retrieved. N0 cases were far more abundant and, thus, we restricted our search in the more recent years of the study period (2015–2016), when a more generous sampling protocol was followed. Follow-up information was obtained from the patients’ files and by contacting them on the phone. The study was approved by the University Hospital of Patras Research Ethics Committee (Protocol Number 195/6.4.2021).

### 2.2. Histopathologic Analysis

**Figure 1:**
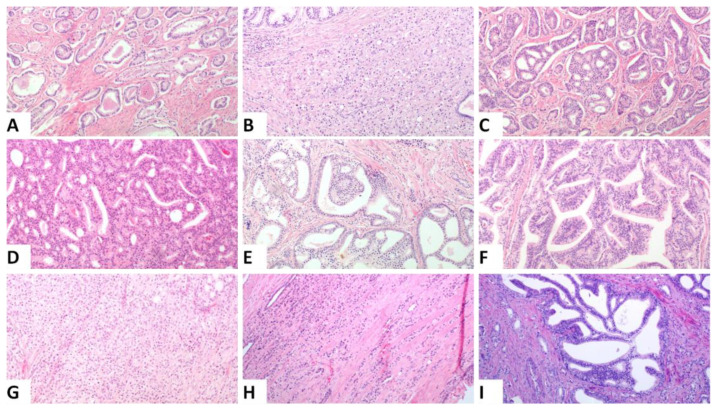
Representative histologic images from the different patterns that were assessed, (**A**). Single glands corresponding to Gleason pattern 3, (**B**). Poorly formed and fused glands, (**C**). Small cribriform formations, (**D**). Large cribriform formations (original magnification ×100), (**E**). Glomeruloid pattern, (**F**). Papillary pattern Images (**B**–**E**) correspond to Gleason pattern 4, (**G**). Solid nests, (**H**). Single cells and cords, Images (**G**,**H**) correspond to Gleason pattern 5, (**I**). Intraductal carcinoma: ((**A**,**B**,**D**,**E**,**G**–**I**): original magnification ×100; (**C**): original magnification ×40; (**F**): original magnification ×200).

All slides from the prostatectomies and lymph nodes were revised by two observers (VT and IMG) and a consensus was reached for each of them. The Gleason Score and PGG (based on 2014 ISUP guidelines and designated as PGG_ISUP2014) [3], pT and pN stage (AJCC 8th edition) [32], presence and extent of extraprostatic extension (EPE) (focal: less than one high power field in less than three sections, vs. non focal: EPE beyond the definition of focal [33]), status of the margins of resection (divided into focal and extensive when positive using the same criteria as in EPE) and tumor volume (percentage of the gland involved) [34] were recorded. Additionally, the percentage of various patterns was separately assessed by visual inspection and recorded. The following patterns were assessed: single glands (corresponding to GG3), fused/poorly formed glands, small cribriform formations, large cribriform formations (>3 times the size of normal glands), glomeruloid formations and papillary pattern (corresponding to GG4), and single cells/cords of cells, solid nests and comedo necrosis (corresponding to GG5). Figure 1 shows representative images from the different patterns that were assessed. The percentage of IDC (in regard to the tumor volume) was also assessed. Assessment of IDC was performed on H&E slides using the criteria established by Guo and Epstein [16] minus the size criterion [14]. Presence of basal cells, corpora amylacea and branched architecture were used as criteria to differentiate IDC from invasive cribriform pattern [35]. Immunohistochemistry for basal cell markers was not performed, unless it had been requested by the diagnosing pathologist when signing out the cases, to mimic routine practice [36]. Cases in which immunohistochemistry would be helpful were noted. Based on the percentage of the different patterns of invasive carcinoma and that of IDC, the PGG according to the ISUP 2019 and the GUPS 2019 guidelines were then calculated, labeled as PGG_ISUP2019 and PGG_GUPS. All parameters mentioned above were assessed for the index tumor focus defined as the focus with the highest T stage. For foci of similar T stage, index focus was defined as the one with the highest PGG. If both PGG and T stage were the same, index focus was defined as the largest one.

### 2.3. Statistical Analysis

Categorical data were summarized using tables, whereas continuously scaled data were summarized with descriptive statistical measures [mean with standard deviation (SD)]. The non-parametric tests, Kruskal Wallis and Mann Whitney, were used to compare the amount of patterns between patient groups. Spearman’s correlation was used to correlate between markers. A chi-square test was used to assess correlations between categorical data. A one-way analysis of covariance was performed to indicate whether IDC was correlated with pathologic characteristics, using PGG_2014 as a covariate. For categorical variables, a binomial logistic regression analysis was performed to account for multiple independent variables. All reported *p* values are two-sided at a significance level of 0.05. Analyses were performed using IBM SPSS Statistics for Windows, Version 25.0 (IBM Corp. Armonk, NY, USA).

## 3. Results

### 3.1. Patients

A total of 129 cases were retrieved. Two approaches were used in specimen submission. In the older specimens (N = 31), the entire apex and base and every other transverse section were submitted, whereas in the rest of the specimens the posterior area of all transverse sections and the anterior area of every other transverse section, along with the entire base and apex, were submitted. Even though not ideal, both methods, and especially the second one used in the majority of our specimens, have shown an acceptable rate of accuracy in terms of grading and staging [37]. Among the 129 cases, 68 cases had one tumor focus, 38 had two foci, 12 three foci and 11 had four foci. Stage, PGG_ISUP2014, EPE and MOR in our patient cohort of the index focus are show in Table 1.

Despite significant efforts, follow-up information was obtained for only 39 patients. Of them, 36 patients had recurred, 10 patients had developed CRPC and 8 had died. Because of the limited data on patients’ survival, further analysis was not performed. 

### 3.2. Detailed Description of the Patterns

Mean percent (and standard deviation) of the different patterns of the index focus in our cohort is shown in Table 2. IDC was seen in 81 cases, a cribriform pattern in 102 cases and a large cribriform in 23 cases. Four cases had only a large cribriform pattern, whereas the rest had both a large and small cribriform pattern. All cribriform formations were considered in total for further analysis (N = 106). Well-formed glands were seen in 106 cases, fused and poorly formed glands in 121 cases, glomeruloid formations in 21 cases, papillary pattern in 18 cases, single cells in 43 cases and solid nests in 41 cases. Comedo necrosis within invasive carcinoma was seen in 21 cases. In one of the cases, IDC carcinoma without invasive tumor was seen in a different area from the index tumor and has been described before [22].

### 3.3. Incorporating IDC into the PGG Results in a Change in a Minority of Cases

**Figure 2:**
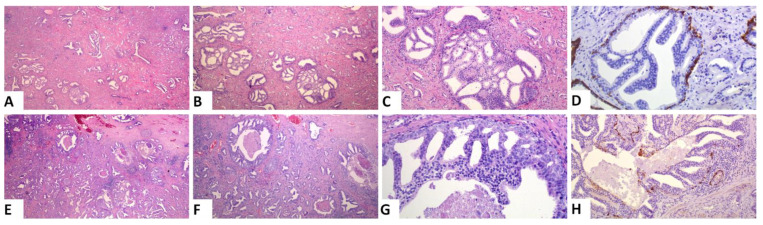
Representative images of the two cases that had a change in PGG when IDC was incorporated into grading: (**A**–**D**) A case with a high amount of IDC and an invasive carcinoma PGG2. Incorporation of IDC into grade will change the grade to 3. (**E**–**H**) A case with extensive IDC with comedo necrosis. Invasive carcinoma is PGG2 (7 3 + 4). Incorporation of IDC into grading will result in a significant upgrade to PGG5. ((**A**,**E**): original magnification ×20; (**B**,**F**): original magnification ×40; (**C**,**G**,**H**): original magnification ×100; (**H**):original magnification ×200; (**D**,**H**): immunohistochemical staining with the basal cell marker 34bE12 cytokeratin).

Based on the amount of the various patterns, we calculated the PGG after incorporating IDC into the grading system (ISUP 2019 guidelines). A change in PGG was noted in 2 of the 129 cases (1.6%). The first case was a PGG_ISUP2014 2 (GS 7, 3 + 4) case that harbored a high amount of IDC and was, thus, changed into PGG_ISUP2019 3 (GS 7, 4 + 3), when IDC was incorporated into the grading system (Figure 2A–C). The patient had a pT3aN1 PCa with non-focal EPE and negative MOR. He experienced a PSA recurrence 2 years after the prostatectomy and is alive 10 years later. The second case was a PGG_ISUP2014 2 (GS 7 3 + 4, with a tertiary pattern 5 due to the presence of comedo necrosis) case with extensive IDC with comedo necrosis that was changed to PGG_ISUP2019 5 (GS 9 4 + 5) (Figure 2D–F). Immunostaining for basal cell markers had been performed at the time of the initial diagnosis of the case to assist with grading. The patient had a pT3aN0 PCa with a large tumor involving 60% of the gland, with non-focal EPE and extensively positive MOR. Early after surgery (9 months) he had a PSA recurrence and, subsequently, he underwent adjuvant radiotherapy. Four years later, he had no other progression of the disease.

### 3.4. Difficulties in Assessing IDC vs. a Cribriform Pattern

**Figure 3:**
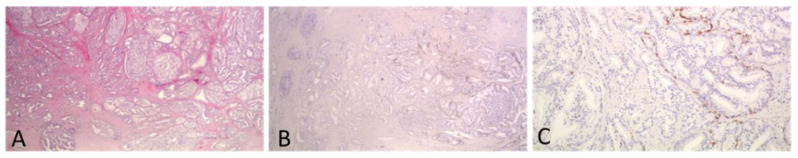
A case with extensive cribriform formations of large caliber. IHC staining for basal cell marker 34bE12 showed that most of the cribriform formations represented IDC. Invasive carcinoma was of a high grade, and thus, PGG would not change if IDC was incorporated into it. ((**A**,**B**): original magnification ×20; (**C**): original magnification ×100).

Difficulty in assessing IDC vs. a cribriform pattern was seen in 8 cases (6.2%). These cases consisted of extensive cribriform formations and there was an uncertainty whether each formation represented a cribriform or IDC focus. In four of them, IHC staining for basal call markers had been performed by the reporting pathologist to assist with the grading and one of those four cases was the one reported above as IDC with comedo necrosis. Immunohistochemistry had not been ordered for the rest of the cases by the reporting pathologist. We assume that this is because no change in the grade of the case would have occurred based on the results of the stains. Representative images from one of these cases are shown in Figure 3. Among the eight cases, four were T3a and four were T3b, four had lymph node metastasis and all but one had non-focal EPE. The number of blocks with a tumor in these cases ranged from 4–7 (mean 5). Interestingly, in only one of these cases there would have been a change in PGG score, if all cribriform formations had been graded. This is the case mentioned in the previous section. For this case, stains had been ordered and reported by the practicing pathologist and revealed that all cribriform formations were indeed IDC, with some of them showing necrosis. At the time of diagnosis of this case, IDC was not included in the grade and was, thus, not graded by the practicing pathologist. However, had IDC been included in the grade, the change would be of great importance as IDC had extensive comedo necrosis and its incorporation in the grading would change the grade from 2 to 5 (Figure 2D–F).

### 3.5. Presence of IDC and Cribriform but Not Comedo Necrosis Are Associated with Adverse Pathologic Parameters

IDC was seen in 81 cases and a cribriform pattern in 106 cases, with 74 cases displaying both patterns, 7 cases showing only IDC and 32 only a cribriform pattern. Statistical analysis revealed that the presence of IDC and cribriform was associated with advanced T stage (*p* < 0.001 for both comparisons), N stage (*p* < 0.001 and *p* = 0.026, respectively), PGG_2014 (*p* < 0.001, for both comparisons), presence and extent of EPE (*p* < 0.001 and *p* = 0.001, respectively) and tumor volume (*p* < 0.001 for both comparisons). No association was noted between each pattern and the status of MOR. In contrast, the presence of comedo necrosis was associated with higher tumor volume (*p* = 0.002), but not T stage, N stage, EPE or status of MOR.

Further analysis was performed in cases with a PGG_2014 of 2 and it was found that the presence of IDC was associated with N stage (*p* = 0.014). A cribriform pattern was correlated with tumor volume (*p* = 0.028) in these cases, but not T or N stage.

Binomial logistic regression analysis that included PGG_2014, T stage, N stage, status of MOR and tumor volume showed that IDC was independently associated with N stage (*p* = 0.016, EXP(B) 0.237, 95% CI: 0.073–0.766) and a cribriform pattern was marginally associated with tumor volume (*p* = 0.047, EXP(B) 1.046, 95% CI: 1.001–1.094).

### 3.6. Amount of IDC but Not Cribriform Pattern Is Associated with Adverse Pathologic Parameters

**Figure 4:**
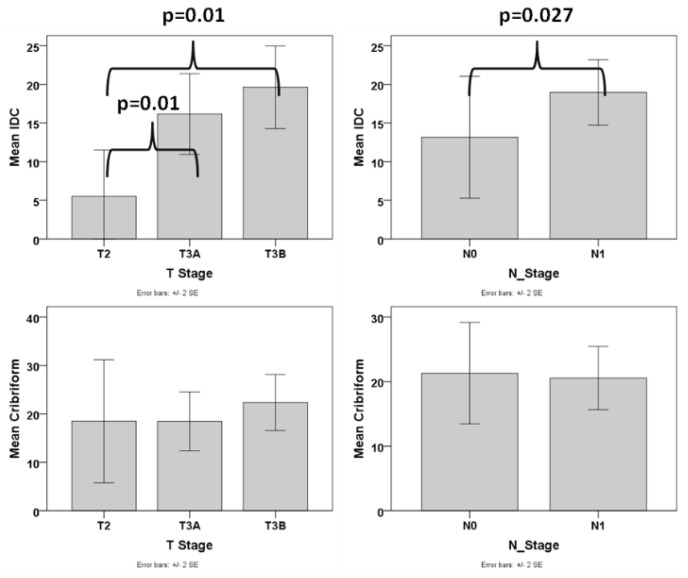
Box plot graphs of the correlation of IDC and Cribriform with T and N stage.

In order to assess whether, besides the presence, also the amount of IDC pattern is an important factor, we excluded the cases that did not have any IDC and analyzed the association of the amount of IDC with various pathologic parameters. We found that the amount of IDC was associated with advanced T stage (difference was statistically significant between T2 and T3a/T3b stage, *p* = 0.01 for both comparisons), N1 stage (*p* = 0.027), presence of EPE (*p* = 0.015) (Figure 4), and higher tumor volume (*p* < 0.001, r = 0.494). The correlation of the amount of IDC with tumor volume (*p* = 0.011) but not T stage, N stage, and EPE was significant when using PGG_ISUP2014 as a covariate. We then analyzed cases with a PGG_2014 of 2 separately and found that the amount of IDC was correlated with advanced T stage (T2 vs. T2a/T3b, *p* = 0.042), EPE (*p* = 0.012) and tumor volume (*p* = 0.011, r = 0.761), despite the small number of patients with PGG2 that had IDC (N = 8).

Similarly, we excluded the cases without any cribriform pattern and analyzed the association of the amount of cribriform with various pathologic parameters. No correlation was found between any of the adverse pathologic parameters and the amount of cribriform pattern (Figure 4).

### 3.7. Following the GUPS 2019 Guidelines Regarding Tertiary Pattern Results in PGG Change in a Number of Cases

GUPS 2019 also has a different definition of a tertiary pattern compared to both ISUP2014 and ISUP2019 guidelines. Following the GUPS 2019 guidelines, PGG was changed in 6 out of 129 (4.7%) cases. Three cases with PGG_ISUP2014 1 and tertiary Gleason pattern 4 were changed to PGG_GUPS 2 (two cases T2 and one T3a, with focal EPE, all N0) and three cases with PGG_ISUP2014 4 and a tertiary Gleason pattern 5 were changed to PGG_GUPS 5 (one T3a and two T3b, one N0 and two N1).

### 3.8. A Significant Difference Exists between ISUP2019 and GUPS2020

A total of 8 cases (6.2%) would be assigned a different PGG when using the ISUP2019 or the GUPS 2020 guidelines, as shown in Table 3.

## 4. Discussion

In the present study, we showed that incorporation of IDC into the grading system changes the grade in a minority of patients, different reporting practices on a tertiary pattern would change the grade in ~5% of the cases, and that depending on the guidelines followed, a different grade would be assigned in >5% of the cases. These findings highlight the need for standardization of grading practices in PCa as this has important implications in the clinical setting. Detailed reporting on the recommendations followed in each case is also needed so that clinicians and patients make informed decisions on management and understand grading or reporting discrepancies among individual pathologists and institutions.

The impact of grading IDC on final grade has been studied before in prostatectomy specimens [35] and biopsies [35,38]. Similar to our study, Rijstenberg et al. showed that tumor grade was changed in 0.6% of the prostatectomy cases when IDC was incorporated into grading [35]. In their series, PGG changed by one point in all cases, whereas in our series a change by three points was seen in one of our cases. The latter has been described by Chen-Maxwell et al., where a change was seen in 23% of the biopsies that exhibited IDC (0.6% of the total number of cases, though data on how many of them were neoplastic were not provided) and one of their cases was upgraded by 3 points. The change was usually due to an increased amount of pattern 4 [35,38] and the presence of comedo necrosis [38], as shown in our study.

Previous studies have used IHC for basal cell markers in a significant proportion of the cases to differentiate between invasive cribriform and IDC [35,38,39]. It has been stated that, the use of IHC in routine settings will pose an additional burden (both financial and in terms of workload) in the already strained pathology departments. This has been used as an argument to support inclusion of IDC into the grading system [15]. Alternatively, it has been proposed to use IHC only in cases in which grading is going to change based on the results of the stains [14]. In this study, we assessed both the number of cases in which there was a difficulty in differentiating IDC from an invasive cribriform pattern and the ones where stains would actually be significant. Even though cribriform formations frequently pose difficulties as to their true nature (in this cohort and in daily practice) [28], this was particularly prevalent in 6% of the cases. However, only in one case (0.8%) there would be a change in the grade if all cribriform formations had been graded. Of note, in this case the change would be significant since IDC was associated with comedo necrosis. However, our results show that the scenario that would require IHC to accurately assign a grade in prostatectomy specimens is not all that prevalent. Still though, the additional load imposed in the department may be high, as these cases usually have multiple blocks that require staining. In addition, the scenario may be more prevalent in biopsies, in which many of the criteria used to differentiate IDC from invasive cribriform carcinoma may not be present [40]. Of note, the use of immunohistochemistry does not guarantee differentiation of IDC from invasive cribriform carcinoma, since the basal cell layer of IDC is fragmented and may not be evident in the plane of the section [41].

Previous studies have shown that the presence of IDC is associated with adverse pathologic parameters and worse prognosis [24,25,26]. In addition, the presence of IDC (and also ductal carcinoma) has been associated with bi-allelic mutations in the genes of proteins involved in DNA repair through homologous recombination (i.e., BRACA2, BRACA1) [42,43]. Thus, according to guidelines, the presence of IDC is an indicator to test the patient for germline mutations in those genes [44]. There is evidence in the literature supporting the incorporation of IDC into grading. Kato et al. showed that prostate carcinomas with IDC had a worse prognosis than PCa of any grade without IDC [45]. This indicates that PGG2 carcinomas with IDC behaved worse than PGG4 and 5 carcinomas without IDC. Similarly, van Leenders et al. showed that a modified PGG that incorporated the presence of IDC (and a cribriform pattern), showed better discriminative value for patients’ survival [46]. In addition, molecular analysis of the cribriform pattern and IDC has revealed that they share genomic instability, including deletions and amplifications in several genes related to aggressive clinical behavior such as loss of *PTEN*, *RB1*, *TP53* and amplification of *MYC* [47]. However, when incorporating IDC into the grading system, as has been proposed by ISUP, it is not only its presence, but also its amount that will influence the grade. Thus, we examined the correlation of the amount of IDC with pathologic parameters and showed that it was significantly associated with adverse pathologic parameters such as advanced T and N stage, presence and extent of EPE and larger tumor volume. In addition, in PGG2 tumors, not only the presence but also the amount of IDC correlated with advanced T stage and larger tumor volume, albeit the number of patients in this analysis was rather small. Taken together, these findings not only highlight the aggressive nature of IDC but also support its incorporation into grading in a quantitative fashion (the cribriform pattern that is incorporated into grading showed similar or less strong associations). A higher prognostic significance of intraductal carcinoma compared to an invasive cribriform pattern present in biopsies has been reported in patients receiving external beam radiotherapy [48]. There is, however, still a long way to go before incorporating IDC into the grade and prospective studies with survival as an endpoint and with a significant number of cases in which the grade would actually change when IDC is incorporated are needed.

Apart from IDC grading, the second major difference observed in the published guidelines was the definition and reporting of tertiary patterns [28]. We are not aware of any study that has shown the impact of this discrepancy. In our study, we showed that in ~5% of our cases the final grade would be different when following the different guidelines provided by GUPS and ISUP regarding the tertiary or minor pattern. Although the prognostic value of a tertiary pattern has been assessed by various studies, the results are limited by the different definitions for a tertiary pattern (ranging from <5% to any amount as long as it is the third most common pattern) and the different endpoints used in various studies [29,49,50,51]. Of note, the results of our study support the 2014 and 2019 ISUP approach, as the presence of a tertiary pattern at least in the low-grade cases, was associated with favorable histologic parameters. However, the number of cases in our study is small for definite conclusions and additional studies are needed to determine the best approach in handling tertiary or minor high-grade patterns in prostatectomy specimens.

Interestingly, the number of cases that would be assigned a different grade when following the different guidelines was not insignificant. In 6% (8 out of 129 cases) a different final grade would be assigned depending on which guidelines were followed by the pathologist. This highlights the potential for confusion when cases graded with a different system are being studied or treated and underscores the need for a consensus grading system. The confusion will be even greater if a pathologist follows the 2019ISUP guidelines in one aspect (i.e., regarding the tertiary pattern) and the GUPS guidelines in another aspect (i.e., regarding the grading of IDC). Thus, a specific comment as to how grading was performed is needed in the reports.

The limitations of our study should be acknowledged and include the retrospective nature of the analysis, the partial (albeit generous in most cases) embedding of the specimens and the lack of immunohistochemical studies for basal cell markers. However, this represents the first study to have addressed the effect of the different grading recommendations in the final grade and the association of the amount of IDC and cribriform patterns with adverse pathologic parameters.

## 5. Conclusions

In conclusion, our study showed that incorporation of IDC into PGG results in a change for a minority of cases, but the change may be significant in some of them. In addition, following the different guidelines issued recently would result in a different grade in a significant number of cases in prostatectomy specimens, highlighting the need for standardization of PCa grading and for additional studies in regard to which PGG has better clinical relevance. In support of IDC incorporation into grading was the finding that not only the presence but also the amount of IDC was associated with adverse pathologic parameters. In addition, the finding that cases with minor pattern 4 had favorable histology argues against inclusion of minor high-grade patterns into PGG. Thus, further refinement and standardization of PCa grading is of paramount importance.

## Figures and Tables

**Table 1:** Pathologic characteristics of the patients.

PGG_ISUP2014	1	2	3	4	5
	8	23	34	17	47
**T stage**	**T2**	**T3a**	**T3b**		
	27	45	57		
**N stage**	**N0**	**N1**			
	54	75			
**EPE**	**Absent**	**Focal**	**Non focal**		
	28	22	79		
**MOR**	**Negative**	**Focal**	**Extensive**		
	69	26	34		

**Table 2:** Distribution of the different patterns.

% of Tumor Volume	IDC	Well Formed Glands	Cribriform	Large Cribiform	Fused/Poorly Formed Glands	Glomeruloid	Papillary	Single Cells/Cords	Solid Nests
**Mean**	11	29	12	5	39	1	3	4	8
**Median**	5	12	5	0	34	0	0	0	0
**Std. Deviation**	15	34	15	14	28	3	11	11	18
**Minimum**	0	0	0	0	0	0	0	0	0
**Maximum**	80	100	75	75	98	20	90	90	95

**Table 3:** Comparison between the two grading recommendations.

Variation		PGG_GUPS					Total
		1	2	3	4	5	
PGG_ISUP2019	1	5	3	0	0	0	8
	2	0	21	0	0	0	21
	3	0	1	34	0	0	35
	4	0	0	0	14	3	17
	5	0	1 *	0	0	47	48
Total		5	26	34	14	50	129

* Denotes a case with a three-point difference in grading.

## Data Availability

Data can be available upon request.

## References

[1] Gleason D.F., Mellinger G.T. (1974). Prediction of Prognosis for Prostatic Adenocarcinoma by Combined Histological Grading and Clinical Staging. J. Urol..

[2] Epstein J.I., Allsbrook W.C., Amin M.B., Egevad L.L. (2005). The 2005 International Society of Urological Pathology (ISUP) Consensus Conference on Gleason Grading of Prostatic Carcinoma. Am. J. Surg. Pathol..

[3] Epstein J.I., Egevad L., Amin M.B., Delahunt B., Srigley J.R., Humphrey P.A. (2016). The 2014 international society of urological pathology (ISUP) consensus conference on gleason grading of prostatic carcinoma definition of grading patterns and proposal for a new grading system. Am. J. Surg. Pathol..

[4] Matoso A., Epstein J.I. (2016). Grading of Prostate Cancer: Past, Present, and Future. Curr. Urol. Rep..

[5] Humphrey P.A., Amin M.B., Berney D.M., Billis A., Cao D., Cheng L., Delahunt B., Egevad L.L., Epstein J.I., Fine S.W., Moch H., Humphrey P.A., Ulbright T.M., Reuter V.E. (2016). WHO Classification of Tumours of the Urinary System and Male Genital Organs.

[6] Epstein J.I., Zelefsky M.J., Sjoberg D.D., Nelson J.B., Egevad L., Magi-Galluzzi C., Vickers A.J., Parwani A.V., Reuter V.E., Fine S.W. (2016). A Contemporary Prostate Cancer Grading System: A Validated Alternative to the Gleason Score. Eur. Urol..

[7] Rubin M.A., Girelli G., Demichelis F. (2016). Genomic correlates to the newly proposed grading prognostic groups for prostate cancer. Eur. Urol..

[8] Kweldam C.F., van Leenders G.J., van der Kwast T. (2019). Grading of prostate cancer: A work in progress. Histopathology.

[9] Montironi R., Cimadamore A., Cheng L., Lopez-Beltran A., Scarpelli M. (2018). Prostate cancer grading in 2018: Limitations, implementations, cribriform morphology, and biological markers. Int. J. Biol. Markers.

[10] Rubin M.A., de La Taille A., Bagiella E., Olsson C.A., O’Toole K.M. (1998). Cribriform carcinoma of the prostate and cribriform prostatic intraepithelial neoplasia: Incidence and clinical implications. Am. J. Surg. Pathol..

[11] Kweldam C.F., van der Kwast T., van Leenders G.J. (2018). On cribriform prostate cancer. Transl. Androl. Urol..

[12] O’Brien C., True L.D., Higano C.S., Rademacher B.L.S., Garzotto M., Beer T.M. (2010). Histologic changes associated with neoadjuvant chemotherapy are predictive of nodal metastases in patients with high-risk prostate cancer. Am. J. Clin. Pathol..

[13] Iczkowski K.A., Torkko K.C., Kotnis G.R., Wilson R.S., Huang W., Wheeler T.M., Abeyta A.M., La Rosa F.G., Cook S., Werahera P.N. (2011). Digital quantification of five high-grade prostate cancer patterns, including the cribriform pattern, and their association with adverse outcome. Am. J. Clin. Pathol..

[14] Epstein J.I., Amin M.B., Fine S.W., Algaba F., Aron M., Baydar D.E., Beltran A.L., Brimo F., Cheville J.C., Colecchia M. (2021). The 2019 Genitourinary Pathology Society (GUPS) White Paper on Contemporary Grading of Prostate Cancer. Arch. Pathol. Lab. Med..

[15] van Leenders G.J.L.H., van der Kwast T.H., Grignon D.J., Evans A.J., Kristiansen G., Kweldam C.F., Litjens G., McKenney J.K., Melamed J., Mottet N. (2020). The 2019 International Society of Urological Pathology (ISUP) Consensus Conference on Grading of Prostatic Carcinoma. Am. J. Surg. Pathol..

[16] Guo C.C., Epstein J.I. (2006). Intraductal carcinoma of the prostate on needle biopsy: Histologic features and clinical significance. Mod. Pathol..

[17] Varma M., Delahunt B., Egevad L., Samaratunga H., Kristiansen G. (2019). Intraductal carcinoma of the prostate: A critical re-appraisal. Virchows Arch..

[18] Samaratunga H., Delahunt B., Yaxley J.W., Johannsen S., Egevad L. (2021). Intraductal Carcinoma of the Prostate: Extreme Nuclear Size Is Not a Diagnostic Parameter. Am. J. Surg. Pathol..

[19] Robinson B., Magi-Galluzzi C., Zhou M. (2012). Intraductal carcinoma of the prostate. Arch. Pathol. Lab. Med..

[20] Khani F., Wobker S.E., Hicks J.L., Robinson B.D., Barbieri C.E., De Marzo A.M., Epstein J.I., Pritchard C.C., Lotan T.L. (2019). Intraductal carcinoma of the prostate in the absence of high-grade invasive carcinoma represents a molecularly distinct type of in situ carcinoma enriched with oncogenic driver mutations. J. Pathol..

[21] Khani F., Epstein J.I. (2015). Prostate Biopsy Specimens With Gleason 3+3=6 and Intraductal Carcinoma. Am. J. Surg. Pathol..

[22] Grypari I.M., Logotheti S., Lazaris A.C., Kallidonis P., Fokaefs E., Melachrinou M., Zolota V., Tzelepi V. (2020). Isolated Intraductal Carcinoma of the Prostate in Prostatectomy Specimens: Report of 2 Cases and Review of the Literature. Int. J. Surg. Pathol..

[23] Montironi R., Zhou M., Magi-Galluzzi C., Epstein J.I. (2018). Features and Prognostic Significance of Intraductal Carcinoma of the Prostate. Eur. Urol. Oncol..

[24] Van Der Kwast T., Al Daoud N., Collette L., Sykes J., Thoms J., Milosevic M., Bristow R.G., Van Tienhoven G., Warde P., Mirimanoff R.-O.O. (2012). Biopsy diagnosis of intraductal carcinoma is prognostic in intermediate and high risk prostate cancer patients treated by radiotherapy. Eur. J. Cancer.

[25] Kimura K., Tsuzuki T., Kato M., Saito A.M., Sassa N., Ishida R., Hirabayashi H., Yoshino Y., Hattori R., Gotoh M. (2014). Prognostic value of intraductal carcinoma of the prostate in radical prostatectomy specimens. Prostate.

[26] Efstathiou E., Abrahams N.A., Tibbs R.F., Wang X., Pettaway C.A., Pisters L.L., Mathew P.F., Do K.-A., Logothetis C.J., Troncoso P. (2010). Morphologic Characterization of Preoperatively Treated Prostate Cancer: Toward a Post-Therapy Histologic Classification. Eur. Urol..

[27] Chen Z., Chen N., Shen P., Gong J., Li X., Zhao T., Liao B., Liu L., Liu Z., Zhang X. (2015). The presence and clinical implication of intraductal carcinoma of prostate in metastatic castration resistant prostate cancer. Prostate.

[28] Smith S.C., Gandhi J.S., Moch H., Aron M., Compérat E., Paner G.P., McKenney J.K., Amin M.B. (2021). Similarities and Differences in the 2019 ISUP and GUPS Recommendations on Prostate Cancer Grading: A Guide for Practicing Pathologists. Adv. Anat. Pathol..

[29] Taguchi S., Morikawa T., Shibahara J., Fukuhara H. (2021). Prognostic significance of tertiary Gleason pattern in the contemporary era of Gleason grade grouping: A narrative review. Int. J. Urol..

[30] Paner G., Srigley J., Pettus J., Giannico G.A., Sirintrapun J., Harik L. (2021). Protocol for the Examination of Prostate Needle Biopsies From Patients with Carcinoma of the Prostate Gland: Case Level Reporting-College of American Pathologists. Shttps://www.cap.org/protocols-and-guidelines/cancer-reporting-tools/cancer-protocol-templates#protocols.

[31] Paner G., Srigley J., Pettus J., Giannico G.A., Sirintrapun J., Harik L.R. (2021). Protocol for the Examination of Radical Prostatectomy Specimens from Patients with Carcinoma of the Prostate Gland-College of American Pathologists. https://www.cap.org/protocols-and-guidelines/cancer-reporting-tools/cancer-protocol-templates#protocols.

[32] Buyyounouski M.K., Choyke P.L., Kattan M.W., McKenney J.K., Srigley J.R., Barocas D., Brimo F., Brookland R., Epstein J.I., Fine S.W., Amin M.B. (2017). Prostate. AJCC Cancer Staging Manual.

[33] Jeong B.C., Chalfin H.J., Lee S.B., Feng Z., Epstein J.I., Trock B.J., Partin A.W., Humphreys E., Walsh P.C., Han M. (2015). The Relationship Between the Extent of Extraprostatic Extension and Survival Following Radical Prostatectomy. Eur. Urol..

[34] van der Kwast T.H., Amin M.B., Billis A., Epstein J.I., Griffiths D., Humphrey P.A., Montironi R., Wheeler T.M., Srigley J.R., Egevad L. (2011). International Society of Urological Pathology (ISUP) Consensus Conference on Handling and Staging of Radical Prostatectomy Specimens. Working group 2: T2 substaging and prostate cancer volume. Mod. Pathol..

[35] Rijstenberg L.L., Hansum T., Hollemans E., Kweldam C.F., Kümmerlin I.P., Bangma C.H., Kwast T.H., Roobol M.J., Leenders G.J.L.H. (2020). Intraductal carcinoma has a minimal impact on Grade Group assignment in prostate cancer biopsy and radical prostatectomy specimens. Histopathology.

[36] Gandhi J.S., Smith S.C., Paner G.P., McKenney J.K., Sekhri R., Osunkoya A.O., Baras A.S., DeMarzo A.M., Cheville J.C., Jimenez R.E. (2020). Reporting Practices and Resource Utilization in the Era of Intraductal Carcinoma of the Prostate. Am. J. Surg. Pathol..

[37] Sehdev A.E.S., Pan C.C., Epstein J.I. (2001). Comparative analysis of sampling methods for grossing radical prostatectomy specimens performed for nonpalpable (stage T1c) prostatic adenocarcinoma. Hum. Pathol..

[38] Chen-Maxwell D., Prendeville S. (2020). Grading of prostate cancer: The impact of including intraductal carcinoma on the overall Grade Group assigned in diagnostic biopsies. Histopathology.

[39] Sehn J.K. (2018). Prostate Cancer Pathology: Recent Updates and Controversies. Mo. Med..

[40] Acosta A.M., Vormittag E., Al Rasheed M.R.H., Sharif A., Mon K.-S., Kajdacsy-Balla A., Mohapatra G. (2018). Comparison of prostatic adenocarcinoma Gleason 5 and intraductal carcinoma of the prostate with tumor necrosis. A morphometric study. Pathol.-Res. Pract..

[41] Varma M. (2021). Intraductal Carcinoma of the Prostate: A Guide for the Practicing Pathologist. Adv. Anat. Pathol..

[42] Risbridger G.P., Taylor R.A., Clouston D., Sliwinski A., Thorne H., Hunter S., Li J., Mitchell G., Murphy D., Frydenberg M. (2015). Patient-derived Xenografts Reveal that Intraductal Carcinoma of the Prostate Is a Prominent Pathology in BRCA2 Mutation Carriers with Prostate Cancer and Correlates with Poor Prognosis. Eur. Urol..

[43] Lozano R., Salles D.C., Sandhu S., Aragón I.M., Thorne H., López-Campos F., Rubio-Briones J., Gutierrez-Pecharroman A.M., Maldonado L., di Domenico T. (2021). Association between BRCA2 alterations and intraductal and cribriform histologies in prostate cancer. Eur. J. Cancer.

[44] Giri V.N., Knudsen K.E., Kelly W.K., Cheng H.H., Cooney K.A., Cookson M.S., Dahut W., Weissman S., Soule H.R., Petrylak D.P. (2020). Implementation of Germline Testing for Prostate Cancer: Philadelphia Prostate Cancer Consensus Conference 2019. J. Clin. Oncol..

[45] Kato M., Hirakawa A., Kobayashi Y., Yamamoto A., Ishida R., Sano T., Kimura T., Majima T., Ishida S., Funahashi Y. (2019). The influence of the presence of intraductal carcinoma of the prostate on the grade group system’s prognostic performance. Prostate.

[46] van Leenders G.J.L.H., Kweldam C.F., Hollemans E., Kümmerlin I.P., Nieboer D., Verhoef E.I., Remmers S., Incrocci L., Bangma C.H., van der Kwast T.H. (2020). Improved Prostate Cancer Biopsy Grading by Incorporation of Invasive Cribriform and Intraductal Carcinoma in the 2014 Grade Groups. Eur. Urol..

[47] Böttcher R., Kweldam C.F., Livingstone J., Lalonde E., Yamaguchi T.N., Huang V., Yousif F., Fraser M., Bristow R.G., van der Kwast T. (2018). Cribriform and intraductal prostate cancer are associated with increased genomic instability and distinct genomic alterations. BMC Cancer.

[48] Tom M.C., Nguyen J.K., Lucianò R., Mian O.Y., Stephans K.L., Ciezki J.P., Smile T.D., Wei W., McKenney J.K., Magi-Galluzzi C. (2019). Impact of Cribriform Pattern and Intraductal Carcinoma on Gleason 7 Prostate Cancer Treated with External Beam Radiotherapy. J. Urol..

[49] Özsoy M., D’Andrea D., Moschini M., Foerster B., Abufaraj M., Mathieu R., Briganti A., Karakiewicz P.I., Roupret M., Seitz C. (2018). Tertiary Gleason pattern in radical prostatectomy specimens is associated with worse outcomes than the next higher Gleason score group in localized prostate cancer. Urol. Oncol. Semin. Orig. Investig..

[50] Pan C.-C., Potter S.R., Partin A.W., Epstein J.I. (2000). The Prognostic Significance of Tertiary Gleason Patterns of Higher Grade in Radical Prostatectomy Specimens. Am. J. Surg. Pathol..

[51] Turker P., Bas E., Bozkurt S., Günlüsoy B., Sezgin A., Postacı H., Turkeri L. (2013). Presence of high grade tertiary Gleason pattern upgrades the Gleason sum score and is inversely associated with biochemical recurrence-free survival. Urol. Oncol. Semin. Orig. Investig..

